# Automated large-scale prediction of exudative AMD progression using machine-read OCT biomarkers

**DOI:** 10.1371/journal.pdig.0000106

**Published:** 2023-02-15

**Authors:** Akos Rudas, Jeffrey N. Chiang, Giulia Corradetti, Nadav Rakocz, Oren Avram, Eran Halperin, Srinivas R. Sadda

**Affiliations:** 1 Department of Computational Medicine, University of California Los Angeles, Los Angeles, California, United States of America; 2 Doheny Eye Institute, Pasadena, California, United States of America; 3 Department of Ophthalmology, University of California Los Angeles, Los Angeles, California, United States of America; 4 Department of Computer Science, University of California Los Angeles, Los Angeles, California, United States of America; 5 Department of Anesthesiology and Perioperative Medicine, University of California Los Angeles, Los Angeles, California, United States of America; 6 Department of Human Genetics, University of California Los Angeles, Los Angeles, California, United States of America; Duke-NUS Medical School, SINGAPORE

## Abstract

Age-related Macular Degeneration (AMD) is a major cause of irreversible vision loss in individuals over 55 years old in the United States. One of the late-stage manifestations of AMD, and a major cause of vision loss, is the development of exudative macular neovascularization (MNV). Optical Coherence Tomography (OCT) is the gold standard to identify fluid at different levels within the retina. The presence of fluid is considered the hallmark to define the presence of disease activity. Anti-vascular growth factor (anti-VEGF) injections can be used to treat exudative MNV. However, given the limitations of anti-VEGF treatment, as burdensome need for frequent visits and repeated injections to sustain efficacy, limited durability of the treatment, poor or no response, there is a great interest in detecting early biomarkers associated with a higher risk for AMD progression to exudative forms in order to optimize the design of early intervention clinical trials. The annotation of structural biomarkers on optical coherence tomography (OCT) B-scans is a laborious, complex and time-consuming process, and discrepancies between human graders can introduce variability into this assessment. To address this issue, a deep-learning model (SLIVER-net) was proposed, which could identify AMD biomarkers on structural OCT volumes with high precision and without human supervision. However, the validation was performed on a small dataset, and the true predictive power of these detected biomarkers in the context of a large cohort has not been evaluated. In this retrospective cohort study, we perform the largest-scale validation of these biomarkers to date. We also assess how these features combined with other EHR data (demographics, comorbidities, etc) affect and/or improve the prediction performance relative to known factors. Our hypothesis is that these biomarkers can be identified by a machine learning algorithm without human supervision, in a way that they preserve their predictive nature. The way we test this hypothesis is by building several machine learning models utilizing these machine-read biomarkers and assessing their added predictive power. We found that not only can we show that the machine-read OCT B-scan biomarkers are predictive of AMD progression, we also observe that our proposed combined OCT and EHR data-based algorithm outperforms the state-of-the-art solution in clinically relevant metrics and provides actionable information which has the potential to improve patient care. In addition, it provides a framework for automated large-scale processing of OCT volumes, making it possible to analyze vast archives without human supervision.

## Author summary

Neovascular exudative Age-related Macular Degeneration (wet AMD) is a late stage of an irreversible eye-disease and a major cause of permanent vision-loss in the United States. Anti-VEGF injections are available to treat this disease, however its efficacy highly depends on early identification of the condition. It is characterized by the presence of fluid inside the retina which can be determined by assessing the presence of specific biomarkers in volumes obtained by Optical Coherence Tomography (OCT) scans performed on the eye. The annotation of structural biomarkers on these volumes is a laborious, complex and time-consuming process, and discrepancies between human graders can introduce variability into this assessment. A previous algorithm called SLIVER-net was shown to accurately identify these biomarkers automatically based on OCT volumes. In this paper we demonstrate on a large dataset that the automatically identified biomarkers can be utilized to predict disease progression in the following 2 years from a baseline visit, enabling early risk assessment and potentially enhancing treatment efficacy, ultimately resulting in an increased quality of life for patients with wet AMD.

## Introduction

Age-related Macular Degeneration (AMD) represents the leading cause of irreversible blindness in subjects older than 55 years of age in developed countries [1]. As the population ages and life expectancy increases, the incidence of the disease is projected to rise [2]. The late stage of the disease is characterized by the presence of geographic atrophy (GA), macular atrophy (MA) or macular neovascularization (MNV) [3–5].

In contrast to atrophic AMD, anti-vascular endothelial growth factor (anti-VEGF) therapy has proven to be effective at reducing vision loss and even improving vision in eyes with neovascular or wet AMD. However, even with consistent treatment, vision loss and progression to atrophy may occur even in eyes with MNV [6,7]. Studies have shown that best visual outcomes are achieved by detecting the neovascular disease activity early and treating before significant visual loss has occurred [8–10].

As a result of this desire to detect disease progression early on, there has been significant effort to identify biomarkers which may predict the development of advanced AMD. Identification of biomarkers has been facilitated by the broad availability of optical coherence tomography (OCT), which has become the dominant imaging technology in ophthalmic clinical practice. Studies evaluating OCT have identified a number of features including high central drusen volume (hcDV), subretinal drusenoid deposits (SDD) and, or reticular pseudodrusen (RPD), intraretinal hyperreflective foci (IHRF), and hyporeflective drusen cores (hDC), which have been shown to be associated with a higher risk for progression to advanced AMD [11–13]. However, identification of these biomarkers requires extensive training and careful examination of the individual B-scans in the OCT volume–this may be challenging in the context of a busy clinical practice and may be susceptible to variability in interpretation among clinicians. Therefore, machine learning algorithms have been developed to automatically detect structural OCT B-scan biomarkers predictive for progression to advanced AMD [14]. By automating the interpretation of OCT volumes, this approach enables low-cost, large-scale studies and analyses of AMD progression while anchoring inferences and conclusions to clinically-relevant biomarkers. However, machine learning approaches in detecting early biomarkers of disease have been only tested in small cohorts, not accounting for heterogeneity in the prediction of the outcome between different environments, settings and populations [15,16].

In the present study, we offer the largest machine learning validation to date of these structural OCT B-scan biomarkers predictive for AMD progression. Our hypothesis is that these biomarkers can be inferred by a machine learning algorithm without human supervision, in a way that they preserve their predictive nature. The way we test this hypothesis is by building machine learning models upon these machine-read biomarkers, and assess their predictive power. Consequently, we also validate the high accuracy with which SLIVER-net automatically detects structural OCT B-scan biomarkers in a large cohort. Our model is not only capable of successfully detecting these structural OCT biomarkers, but also able to predict future AMD progression and prognosis, which may impact clinical decision making. First, we explore the ability of the automated approach to *predict* future conversion to exudative AMD within 2 years from the baseline OCT. Then, we apply our approach to diagnosis, showing that machine-read OCT features are also informative for determining the current disease status. Our approach is able to significantly improve predictive models which consider only the currently available risk factors, and are developed using data from smaller cohorts with less population heterogeneity.

## Results

Machine-read OCT features were evaluated for their clinical utility relative to currently known risk factors contained within the electronic health record using a predictive modeling framework. These features were evaluated in their ability to predict conversion to exudative AMD as well as diagnosis of current exudative AMD.

### Predicting future conversion to Exudative AMD

Using machine-read OCT B-scans features and EHR-derived risk factors together in logistic regression models (*combined*), we were able to successfully predict exudative AMD conversion within two years with an area under the ROC curve (AUROC) of 0.82 (95% confidence interval (CI): 0.78, 0.85) and area under the Precision Recall Curve (AUPRC) of 0.49 (95% CI:0.41, 0.57).

**Fig 1:**
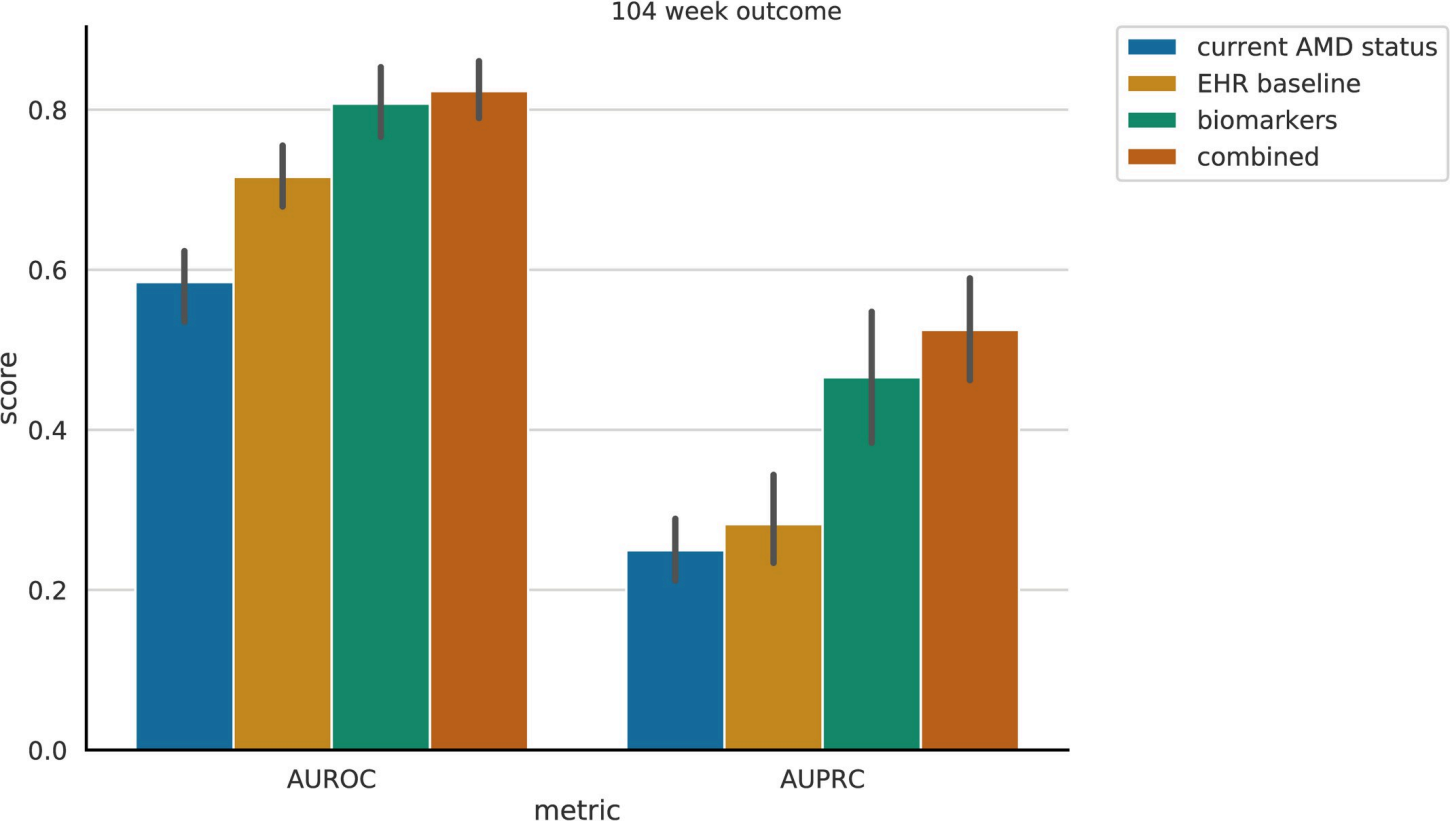
Exudative AMD prediction performance. Left: Areas Under the Receiver Operating Characteristic (ROC) curve. Right: Areas Under the Precision-Recall (PR) curve. Each bar represents the performance utilizing a different set of features (see legend). Error lines represent the 95% confidence intervals, computed using bootstrapping.

**Fig 2:**
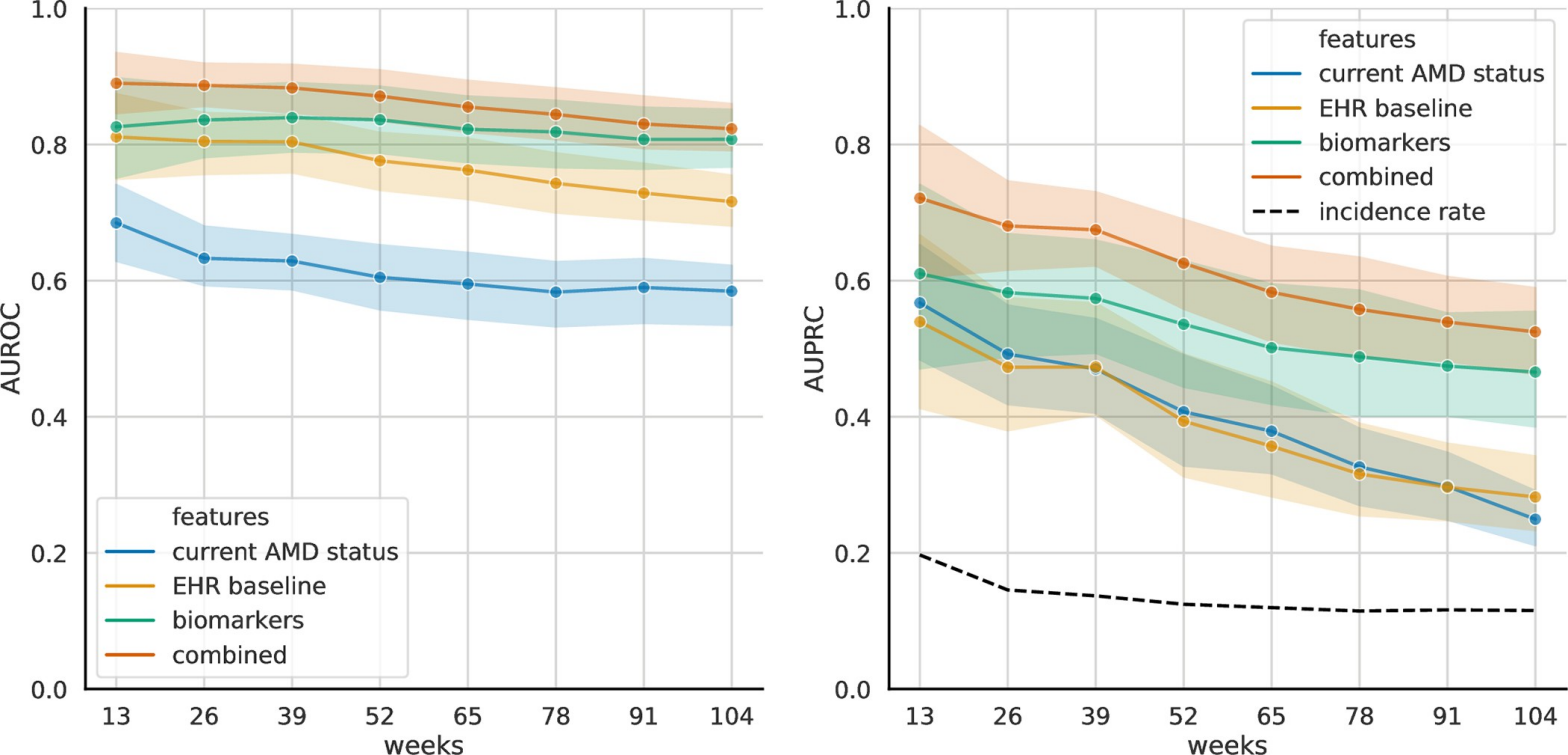
Prediction of exudative AMD Conversion by the 2-year model, evaluated at different time frames. L*eft*. Area under the ROC curve (AUROC) as a function of prediction time frame. *Right*. Area under the Precision-Recall curve (AUPRC) as a function of prediction time frame. 95% Confidence intervals were computed using bootstrapping.

Relative to the EHR-derived features of age, sex, race, smoking status, and comorbidities, the addition of machine-read OCT B-scans features resulted in significantly improved predictive performance in terms of AUROC and AUPRC (see Fig 1). The trivial model (*current AMD status*) utilizing only the presence of dry AMD at the time of examination and the time to the next examination, yielded an AUROC of 0.57 (95% CI: 0.54, 0.60) and AUPRC of 0.21 (95% CI: 0.18, 0.24). With added EHR-derived features and comorbidities (EHR baseline), the performance increased to AUROC of 0.72 (95% CI: 0.69, 0.74) and AUPRC of 0.25 (95% CI: 0.22, 0.28). The machine-read OCT B-scan features (*biomarkers*) were also by themselves highly predictive of exudative AMD conversion (Figs 1 and 2; biomarkers) yielding AUROC of 0.80 (0.78, 0.82) and AUPRC of 0.46 (0.41, 0.50).

Patients could have converted to exudative AMD at any point during the two-year window evaluated. To observe predictive performance over time, the above analysis was repeated at 3-month intervals. When additional models were trained for their ability to predict exudative AMD conversion within 3, 6, 9, 12, 15, 18, 21, and 24 months, up to within two years, we observed the general trend that AUROC was more stable across time periods with a 0.1 decrease in mean (0.9 (0.78, 0.99) @ week 13 -> 0.8 (0.78, 0.82) @ week 104), while there was a general decrease in AUPRC (0.81 (0.63, 0.96) @ week 13 -> 0.55 (0.34, 0.71) @ week 104) with a 0.26 decrease in mean, for the combined model (S5 Fig). To assess whether it is beneficial to train different models for different time frames, the 2-year model was separately evaluated on different time frames (Fig 2). No significant drop in performance was observed. The presence of the biomarkers appeared to be more indicative of imminent exudative AMD conversion.

Table 1 reports a detailed view of all performance metrics for the full model at different operating thresholds for 26 and 104 weeks.

**Table 1:** Performance metrics of the combined prediction model for a timeframe of 26 and 104 weeks. Results using a threshold selected for high sensitivity (>80%), a threshold for high specificity (>90%), and one for a balanced case are presented.

Metric	26 weeks			104 weeks		
Threshold	Balanced	High sensitivity	High specificity	Balanced	High sensitivity	High specificity
False Negative Rate	0.26 (0.17, 0.35)	**0.14 (0.07, 0.24)**	0.43 (0.32, 0.54)	0.29 (0.25, 0.35)	**0.14 (0.1, 0.19)**	0.46 (0.39, 0.54)
False Positive Rate	0.18 (0.15, 0.22)	0.35 (0.28, 0.44)	**0.08 (0.06, 0.1)**	0.23 (0.2, 0.25)	0.44 (0.35, 0.52)	**0.01 (0.09, 0.10)**
Negative Predictive Value	0.95 (0.93, 0.96)	**0.97 (0.94, 0.98)**	0.93 (0.91, 0.94)	0.95 (0.94, 0.96)	**0.97 (0.96, 0.98)**	0.94 (0.93, 0.95)
Positive Predictive Value	0.40 (0.33, 0.48)	0.29 (0.23, 0.36)	**0.55 (0.46, 0.65)**	0.29 (0.25, 0.33)	0.20 (0.17, 0.25)	**0.43 (0.36, 0.47)**
Sensitivity	0.74 (0.65, 0.83)	**0.86 (0.76, 0.93)**	0.57 (0.46, 0.68)	0.71 (0.65, 0.76)	**0.86 (0.81, 0.90)**	0.55 (0.46, 0.61)
Specificity	0.82 (0.78, 0.85)	0.65 (0.56, 0.72)	**0.92 (0.9, 0.94)**	0.77 (0.75, 0.80)	0.56 (0.48, 0.66)	**0.91 (0.90, 0.92)**

### Analysis of the predictive utility of individual biomarkers

In order to assess whether some of the biomarkers are more predictive than others, a comparative analysis was conducted in the following way. Feature sets were recombined to yield 8 additional feature sets: the feature sets current AMD status and EHR baseline were expanded with each feature individually (biomarker-SDD: current AMD status + [SDD], ehr-SDD: EHR baseline features + [SDD], etc). We conclude that no biomarker was significantly more predictive than the rest, based on our dataset (Table 2). Additionally, although the added predictive utility of EHR baseline features was not significant either, their addition affected AUPRC more than AUROC.

**Table 2:** Performance metric comparison of models built upon the trivial feature set and a single biomarker (Without EHR), and the EHR baseline feature set and a single biomarker (With EHR).

	Without EHR		With EHR	
Biomarker	AUROC	AUPRC	AUROC	AUPRC
hDC	0.78 (0.68, 0.88)	0.34 (0.23, 0.48)	0.78 (0.70, 0.86)	0.38 (0.31, 0.45)
SDD	0.81 (0.71, 0.90)	0.44 (0.24, 0.66)	0.81 (0.75, 0.89)	0.49 (0.35, 0.66)
HighDrusenVol	0.81 (0.72, 0.91)	0.49 (0.30, 0.70)	0.82 (0.77, 0.90)	0.52 (0.40, 0.68)
RPD	0.80 (0.70, 0.89)	0.43 (0.29, 0.59)	0.79 (0.72, 0.87)	0.46 (0.39, 0.58)
HRF	0.81 (0.73, 0.90)	0.48 (0.26, 0.69)	0.82 (0.78, 0.91)	0.52 (0.38, 0.68)

### Analysis of model weights

**Fig 3:**
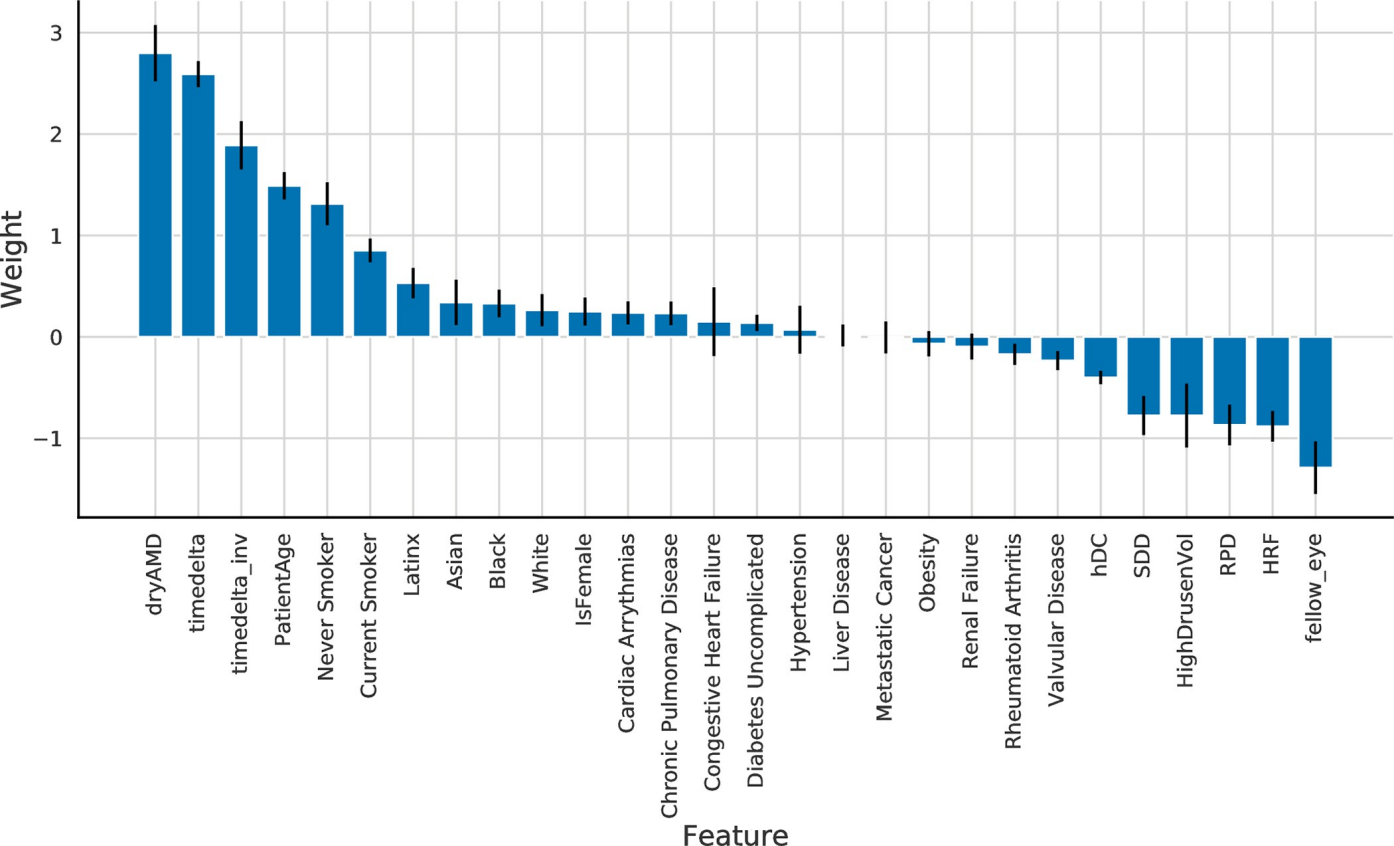
Model weights for all features in the fitted combined model. Black error bars indicate standard deviation of values obtained by fitting the model to bootstrapped subsets of the cohort.

After fitting the logistic regression models, the calculated coefficients were saved to analyze how different features relate to model outcome (Table 3). Fig 3 indicates that the most informative predictors were age, the biomarkers, and the time to a next visit (timedelta). Observing this for age and timedelta are expected; even the name reflects that the disease is age-related, and timedelta serves as the time component for progression prediction. The valuable observation we made here is the large weights of the biomarkers which shows that their presence is associated with disease progression. S1 Fig shows model weights for the biomarkers in the biomarkers prediction model. S2 Fig features a heatmap indicating pairwise feature correlations.

**Table 3:** 95% confidence intervals and standard deviation of values obtained by fitting the model to bootstrapped subsets of the cohort. Feature weights of the combined model.

	mean	2.50%	97.50%	std
hDC	-0.43	-1.081	0.221	0.32
SDD	0.718	0.067	1.481	0.364
HighDrusenVol	1.622	1.09	2.179	0.278
RPD	0.996	0.321	1.754	0.355
HRF	1.918	1.184	2.597	0.353
PatientAge	2.164	1.394	2.983	0.417
Never Smoker	-0.298	-0.628	0.062	0.178
Current Smoker	0.207	-0.847	1.094	0.492
Latinx	-0.042	-0.711	0.615	0.328
Asian	-0.119	-0.68	0.416	0.279
Black	-0.733	-1.439	-0.112	0.352
White	0.24	-0.128	0.696	0.217
IsFemale	-0.001	-0.353	0.341	0.174
Cardiac Arrythmias	0.317	-0.137	0.781	0.231
Chronic Pulmonary Disease	-0.006	-0.542	0.532	0.279
Congestive Heart Failure	0.155	-0.54	0.806	0.337
Diabetes Uncomplicated	-0.314	-0.723	0.083	0.21
Hypertension	-0.147	-0.532	0.215	0.193
Liver Disease	0.162	-0.414	0.673	0.275
Metastatic Cancer	-0.62	-1.267	0.087	0.332
Obesity	0.5	-0.061	0.977	0.256
Renal Failure	0.214	-0.235	0.659	0.231
Rheumatoid Arthritis	-0.593	-1.354	0.124	0.391
Valvular Disease	0.372	-0.445	1.14	0.389
dryAMD	-0.483	-0.896	-0.076	0.206
timedelta	1.114	0.53	1.726	0.295
timedelta_inv	-0.061	-0.409	0.44	0.23

### Large scale validation of machine-read OCT features for diagnosis

**Fig 4:**
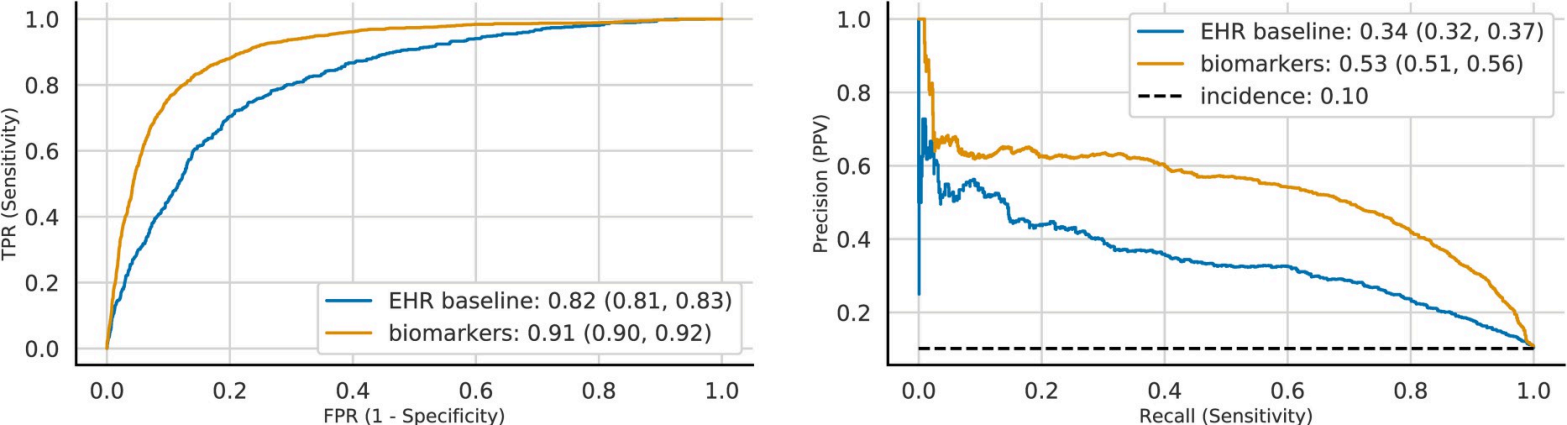
Automated diagnosis of exudative AMD. Curves correspond to models trained on different feature sets (see legend). *Left*. Receiver Operating Characteristics (ROC). *Right*. Precision-Recall curve (PRC). 95% Confidence intervals were computed using bootstrapping. Baseline model utilizes EHR-derived features and risk factors, the biomarker model includes machine-read OCT biomarkers too.

Although these structural OCT B-scan biomarkers are expected to be predictors of AMD progression, and not biomarkers upon which to base a diagnosis, based on the association between these biomarkers and disease severity described by [11], as a validation experiment, we applied the same logistic regression framework -using the same features- in order to diagnose the current eye with exudative AMD. EHR and machine-read OCT B-scan features were used as input features to diagnose exudative AMD. We observed that relative to the EHR-derived features of age, sex, race, smoking status, and comorbidities, which achieved diagnostic performance of AUROC 0.82 (95% CI: 0.81, 0.83) and AUPRC 0.34 (95% CI: 0.32, 0.37), the addition of machine-read OCT B-scan features resulted in significantly improved diagnostic performance in terms of both AUROC [0.91 (95% CI: 0.90, 0.92)] and AUPRC [0.53 (95% CI: 0.50, 0.56)] (Fig 4). This improvement, based on the addition of machine-read OCT B-scan features, was consistent with a clinically validated scoring system [11], in which the presence of SDD, IHRF, and hcDV were associated with higher disease severity and progression.

### Considering AMD status of fellow eye as a feature

The added predictive and diagnostic utility of the AMD status of the fellow eye was assessed.

In the prediction task, adding this feature did not improve predictive power for any of the feature groups (the largest observed increase being 5.1% in the mean AUPRC value for the current AMD status model, with largely overlapping confidence intervals) (S3 Fig).

In the diagnosis task we observed a significant increase in AUROC values between the EHR baseline diagnostic model and its counterpart with fellow eye status: AUROC increased from 0.82 (0.81, 0.83) to 0.87 (0.86, 0.88). The increase for the biomarkers model was present, but significant. However, a significant increase in AUPRC was observed for both models: for the EHR baseline, AUPRC increased from 0.34 (0.32, 0.37) to 0.46 (0.44, 0.49), and for the biomarkers model it increased from 0.53 (0.50, 0.56) to 0.61 (0.58, 0.63) (S4 Fig).

## Discussion

In this study we provide the first large-scale validation of machine-read structural OCT B-scan biomarkers for AMD progression. We do so by utilizing a deep learning method, SLIVER-net, which was trained to identify these biomarkers from OCT volumes. We show that regression models using these biomarkers do indeed predict AMD progression, thus validating not only the accuracy of SLIVER-net, but also the generalizability of these previously proposed structural OCT B-scan biomarkers, and the fact that they can be accurately inferred by a machine learning algorithm without human supervision. The prediction was implemented using a cross validation approach across 15000 OCT volumes collected from nearly 4200 patients.

To validate the utility of machine-read OCT B-scan biomarkers, we automatically predicted conversion to exudative AMD from nonexudative AMD. The automated assessment of conversion to exudative AMD was based on EHR data and OCT B scans volume data from subjects evaluated at ophthalmology clinics affiliated with a large academic hospital during 2018. The outcome (conversion to exudative AMD) was explored using several models and considering the following covariates: current AMD status, EHR-derived risk factors and comorbidities, and structural OCT B-scan biomarkers for progression of AMD. The ability of logistic regression models, trained to predict future conversion to exudative AMD, improved when adding AMD progression biomarkers to comorbidity features and demographic risk factors. Within a minimum of 3 months to a maximum of 2 years, logistic regression models trained with machine-read biomarkers performed with an AUROC of 0.82 (95% CI: 0.78, 0.85) and AUPRC of 0.49 (95% CI:0.41,0.57). This validation approach not only proved to be successful, but provided us with a clinically useful approach, which offers an early warning for the subset of patients identified as having a higher risk of AMD progression. Particularly, our study was performed on 4182 patients, while the largest study to date validating these biomarkers [13] included only 501 patients. De Fauw et al published about the ability of a deep learning algorithm to identify referral-warranted retinal diseases using structural OCT volumes from real-world practices, with a performance similar to human experts [17]. Although in this study the authors used [17] a dataset larger (N = 7,621) than ours, an important distinction is that our dataset included clinically more relevant annotations, using the AMD-related high risk biomarkers. We observed that diagnosis based on these biomarkers outperforms previously reported performance by deep learning approaches.

For a human grader to assess presence of these biomarkers, each B-scan or section in the OCT volume would need to be individually scrutinized which can lead to measurement biases, inter-grader variability, and could take several minutes which is a challenge in a busy clinical practice. For these reasons, in real-world ophthalmic practice, the assessment of these biomarkers on OCT volumes is not yet part of the usual routine in clinical practice. Therefore, the validation of automated OCT annotations using machine learning algorithms is beneficial with the purpose to validate structural OCT features associated with a high-risk for progression to advanced AMD.

Exudation in eyes with macular neovascularization secondary to AMD appears in eyes with the late stage of the disease. The detection of fluid (exudation) at different levels within the retina (intraretinal, subretinal, sub-retinal pigment epithelium) defines the presence of disease activity. The advances in retinal imaging and the introduction of OCT technology have been transformative in the diagnosis, management and follow-up of eyes with exudative AMD, allowing the detection of fluid with high resolution and high precision. Of note, the exudative form of AMD can be successfully treated with anti-VEGF therapy, [4] and it has been established that earlier treatment is associated with better visual outcomes [18]. Therefore, there has been increasing interest in intervening at earlier stages of the disease. A number of studies have identified several high-risk biomarkers on structural OCT B-scan, such as intraretinal hyperreflective foci, subretinal drusenoid deposits, drusen with hyporeflective cores and high central drusen volume, which appear to be associated with a higher risk of progression from intermediate to late AMD [11–13,19–20]. Our group has previously investigated the utility of SLIVER-net in automated detection of these high-risk biomarkers in a small annotated OCT dataset with good performance, sometimes better than retina specialists [14]. In this study, we used these AMD progression biomarkers to predict the conversion to exudative AMD.

Recent work has also applied deep learning to raw OCT volumes to predict 6-month wet AMD conversion in the fellow eye when a patient already had wet AMD in one eye [21]. In the fellow eye of patients who already had exudative AMD, we performed a post-hoc analysis on model performance. The model described by [21] was selected for comparison due to pursuing the same goal—predicting progression to wet AMD. The populations are comparable, with only a difference in the inclusion criteria by [21]. Namely, they included (eyes of) patients with a positive diagnosis for wet AMD in the fellow eye. Being an important distinction, for the purposes of comparison we used a subset of our cohort, having applied the same inclusion criteria regarding the AMD status of the fellow eye. The presented results in the comparison table (Table 4) were obtained on this filtered subset. For the scenario of predicting deterioration in 6 months [21], reported an AUROC of 0.745 and an AUPRC of 0.123 on their test set. Our model reached a mean AUROC of 0.847 (0.716, 0.98) and mean AUPRC of 0.745 (0.539, 0.951) using the same cohort inclusion and exclusion criteria on our dataset. When binarizing their predictions to optimize for a high specificity around 90%, their model reportedly achieved 34% sensitivity and 9.6% false positive rate. Our model, which utilizes machine-read OCT B-scan biomarkers, yielded a 63% (36.7%, 87.5%) sensitivity and a13.5% (7.8%, 19.8%) false positive rate along the same optimization approach. They did not report positive predictive value (PPV) for the model, but in their paper, they included PPV metrics for three retinal specialists and three optometrists. Our model performed on par with them—every clinician’s performance (lowest reported: 18%; highest reported: 36.5% [21]) was within or below our model’s confidence intervals (Table 1, ‘26 weeks’). Our proposed model significantly outperformed the previous algorithm using similar data inclusion criteria (Table 4) in means of FPR, sensitivity (with fixed specificity), and specificity (with fixed sensitivity).

**Table 4:** Performance metric comparison of our combined model and reported performance metrics of the model proposed by [21] using the same selection criteria. *Question marks indicate values not clearly reported.

	Threshold	False Positive Rate	Sensitivity	Specificity
Yim et al.[21]	High sensitivity	0.434	> = 0.8?*	0.55
	High specificity	0.096	0.34	> = 0.9?*
Combined Model	High sensitivity	0.203 (0.129, 0.3)	0.704 (0.444, 0.948)	0.797 (0.7, 0.871)
	High specificity	0.135 (0.078, 0.198)	0.63 (0.367, 0.875)	0.865 (0.802, 0.922)

We found that the machine-driven annotation was able to accurately predict the onset of exudative AMD within two years from the “baseline visit” at which the OCT was acquired.

The importance of predicting the conversion to exudative AMD within 2 years is that it can impact the development of follow-up and monitoring schedules for a patient and for potentially selecting a higher-risk group of patients who may benefit from more expensive home-monitoring strategies [22–24]. A personalized monitoring approach could potentially allow earlier detection of these patients, thereby leading to earlier therapeutic intervention and better visual outcomes.

Lei et al. showed that the presence of these biomarkers were associated with higher AMD disease severity and progression [11]. We attempted to verify that finding by attempting to “impute” the diagnosis or no-diagnosis of wet AMD. We showed that the use of the SLIVER-net OCT biomarkers significantly increased diagnostic accuracy, which is consistent with the findings of [11].

We note that our study has its limitations. Specifically, patients represented in our dataset visited ophthalmic clinics due to a scheduled check-up or an existing complaint or condition, and thus the selection of the patients may affect the generalizability of these results to the general population. However, since our work concentrates on the validation of established biomarkers that have been shown to be predictive of AMD progression in similar datasets [11,13], we do not expect this limitation to be particularly problematic, however we note that additional replication studies would be useful to further validate the biomarkers in the future.

Two additional limitations are the lack of data regarding any external diagnosis the patients might have received, and the right-censored nature of the dataset—observations are limited to a specific time window. To address these issues without reframing the study as survival analysis, we implemented the following design. Data points for the prediction task are technically not the individual exams, rather pairs of exams. For a given time window t, the class label of “Progression happened in t time.” for a quadruple (exam_date1, wetAMD1, exam_date2, wetAMD2) is 1 if wetAMD2 is 1 and the time difference between exam_date1 and exam_date2 are less than t (note that wetAMD1 in this set is always 0, it was mentioned explicitly for clarity). Therefore, it could have not happened that a patient received a positive diagnosis elsewhere, and was considered as a negative case by the algorithm (assuming no false negative diagnoses made by the clinicians).

Our study also has a few strengths. First, the machine learning algorithms have been trained and tested on a large cohort. We have performed a large-scale automatic validation of these previously established biomarkers, validating not only the biomarkers, but their automatic identification as well. Furthermore, we have provided evidence that automatic detection of structural OCT B-scan biomarkers using machine learning can be of value in predicting exudative AMD. The algorithm has the ability to provide automated annotation of these biomarkers on OCT volumes with high precision and feasibility, avoiding the laborious manual inspection or annotation of all the OCT B-scans. Also, considering the challenges associated with implementing and deploying separate models for different time horizons, the 2-year model was separately evaluated on different time frames. Since no significant drop in performance was observed, it is reasonable to assume that the model can successfully utilize the provided timedelta feature. Thus, we determined that it is sufficient to deploy a single model across different time frames.

In conclusion, we demonstrate on a large dataset that a machine learning algorithm can automatically annotate OCT volumes with high-risk structural OCT B-scan biomarkers of AMD progression with high accuracy. These annotations can be used to predict conversion to exudative AMD in eyes with nonexudative AMD with good performance, providing an impactful example of how machine learning has the ability to enhance patient care.

## Methods

### Study design and dataset

The study was conducted in compliance with the Declaration of Helsinki and approved by the UCLA Institutional Review Board (IRB, Ocular Imaging Study; Doheny–UCLA Eye Centers).

The dataset consisted of 14,615 OCT volumes collected from 4,182 patients at affiliated Ophthalmology clinics during 2018 and corresponding electronic health record data for these visits including demographics, AMD status, and comorbidities (see Table 5). OCT volumes were obtained by the Spectralis OCT device (19 B-scans, 20x20 degree centered on the fovea). A single volume for each (exam date, patient, eye) triplet was included in the study. Volumes collected during the same encounter and corresponding to the same eye were aggregated, selecting the maximum measured value for each biomarker on that date. It should be noted that since the dataset in this study was selected from a specific time frame, progression-wise the data is right-censored. Examination in a survival analysis framework is in the scope of future work.

**Table 5:** Descriptive statistics of the dataset.

	Patients	Eyes	OCT volumes
Total	4,182	8,075	14,615
Prediction dataset	1807	2615	4915
Current Wet AMD (%)	462 (11.0%)	636 (7.9%)	1,486 (10.2%)
No Wet AMD w/n 2 years	3,478	7,134	12,523
New Wet AMD w/n 6mo	161	203	350
New Wet AMD w/n 12 mo	203	250	456
New Wet AMD w/n 18 mo	225	281	535
New Wet AMD w/n 24 mo	242	302	606
Age (SD)	66.45 (16.81)	79.10 (8.43)	81.57 (10.25)
Female	53.8%	58.7%	57.9%
Never Smoker	65.0%	71.9%	54.1%
Current Smoker	2.8%	1.8%	4.6%
Latinx	8.9%	8.6%	6.7%
Asian	11.2%	16.7%	9.0%
Black	4.7%	1.8%	1.6%
White	66.4%	67.9%	72.9%

### EHR-derived features and outcomes

AMD status, demographics, and comorbidities were extracted from the electronic health records.

For each eye and visit, the presence of exudative (wet) AMD was defined using the ICD-10 code H35.32XX. The demographic factors extracted were age, sex, race, ethnicity, smoking status [25]. Comorbidities were defined using the CMS [26]: cardiac arrhythmias, chronic pulmonary disease, congestive heart failure, diabetes (uncomplicated), hypertension, liver disease, metastatic cancer, obesity, renal failure, rheumatoid arthritis, valvular disease. All these clinical and demographic data were treated as dichotomous variables (presence/absence).

### Automated quantification of AMD-related biomarkers

SLIVER-net [14] was used to automatically annotate OCT B-scan volumes for the following machine-read structural OCT AMD risk-progression biomarkers: high central drusen volume (hcDV), subretinal drusenoid deposits (SDD) and, or reticular pseudodrusen (RPD), intraretinal hyperreflective foci (IHRF), and hyporeflective drusen cores (hDC). The likelihood of each biomarker being present was represented as a score between 0 and 1. OCT B-scan volumes which we could not link to the EHR were not included in this analysis. Since not all OCT B-scan volumes consisted of the same number of slices, only volumes with at least 19 slices were utilized. Volumes with more than 19 slices were downsampled uniformly.

SLIVER-net was developed using the dataset described in [27] of 4,686 patients, and the Amish Eye Study dataset [28] of 1,007 subjects whose imaging data was manually annotated by clinician experts. The model’s performance was compared to these human expert graders [14], and it was found that SLIVER-net overperformed all clinician experts in identifying subretinal drusenoid deposits (SDD), and it overperformed 2 out of 3 clinicians in identifying intraretinal hyperreflective foci (HRF). Human graders identified hyporeflective drusen cores (hDC) with higher accuracy, however, SLIVER-net predicted high central drusen volume (HighDrusenVol) and reticular pseudodrusen (RPD), something human experts would have needed additional imaging modalities or software analytical tools in order to do.

### Time to a next visit

The time between two visits in the dataset was named timedelta. It was determined by selecting a baseline visit for each patient’s eye during which their eye’s condition had not progressed to wet AMD, and pairing it up with all following visits as candidate future visits. Then, if the patient’s eye status progressed to wet AMD, the earliest visit with the positive diagnosis was selected as the future visit. If the patient’s eye condition did not progress in the study period, a future visit was selected randomly. The feature timedelta was computed as the time difference between the baseline visit and the future visit.

The appropriate time until the next visit is considered by the clinician on a case-by-case basis. This time can be as short as 3 months, or as infrequent as 12 months. It is important to note however, that the timdelta feature in our dataset is not the same as the follow-up time determined by the physician—the exam-pairs in our prediction dataset were selected as described above. In our dataset, the mean timedelta was 408.9 days (SD 266.6). Since these times are on the several months-scale, it could be argued that time availability for a follow-up visit is not a relevant parameter, as on this scale these follow-ups are scheduled well ahead of time and are rescheduled in a timely manner should circumstances warrant.

An additional feature timedelta_inv = 1/timedelta was also added.

### Analyses

We used an 8-fold out-of-sample prediction framework in order to evaluate the predictive utility of the machine-read OCT biomarkers relative to EHR-derived features and risk factors for two tasks: 1) predicting conversion to future exudative AMD, and 2) diagnosis of current exudative AMD. We constructed several candidate feature sets, consisting of machine-read OCT and EHR-derived features and compared prediction performance for models trained using the different feature sets. All analyses were performed using Python, particularly the Scikit-learn [29] and Statsmodels [30] packages.

### Predicting future conversion to Exudative AMD

Logistic regression models were trained on different feature sets in order to predict future conversion to exudative AMD. This analysis was limited to OCT volumes of eyes which did not already exhibit exudative AMD (2615 eyes, 1807 patients). For patients who developed exudative AMD, the earliest appearance of the corresponding ICD-10 code was recorded as the conversion date.

We applied logistic regression analyzes to predict future conversion to exudative wet AMD based on our extracted features. EHR and machine-read OCT B-scan features were used as input features to predict a future diagnosis of exudative Wet AMD. We compared four different combinations of feature groups: 1) the *current AMD status* model used only the current AMD status and time to a next visit (described above); 2) the EHR baseline model used the EHR demographic and comorbid risk factors as well as the time to the next visit; 3) the *biomarkers* model used *only* the machine-read OCT B-scan biomarkers, and 4) the *combined* model incorporated all the features available. This analysis was repeated for time horizons ranging from three to 24 months.

Following the threshold optimization procedures outlined in [21], two operating thresholds were determined such that the model was expected to achieve 80% sensitivity and 90% specificity, respectively. Additionally, to assess how the model performs when they are optimized together instead of independently, we included a threshold for a balance of sensitivity and specificity by finding a threshold which maximizes true positive rate while minimizes false positive rate, i.e. finding a point on the ROC curve close to the top left corner.

We acquired performance metrics in the following manner: in one round of cross-validation we split the data set to train- and validation sets with a ratio of 7:1 in a way that the two sets were disjoint on the patient-level. The logistic regression model was trained on the train set, after which it was used to generate predictions on the same train set. Based on the performance metrics of this prediction, 3 operating thresholds (balanced, high sensitivity, high specificity) were determined. Then the trained model generated predictions between 0 and 1 for the validation set, and predictions were binarized according to the thresholds. From the binarized predictions the rest of the performance metrics could be calculated. Validations were performed for eight rounds (i.e. 8-fold cross validation). To describe the cross-validation methodology in detail:

1. Data gets split to 8 disjoint groups on the patient level
2. 8-fold cross-validation:
  - Model gets trained on 7 folds
  - Threshold is determined on the same 7 folds
  - Predictions generated for the 8th fold
  - AUROC and AUPRC values are calculated for the 8th fold
  - 8th fold predictions, thresholds, AUROC and AUPRC values are saved:
    - Results:
      1. predictions: [predi, 1, predi, 2, …, predi,|slice8|],
      2. thresholds: [thr_opti, thr_opt_sensi, thr_opt_speci],
      3. auroc: AUROCi,
      4. auprc: AUPRCi.
  - 8th fold predictions are binarized based on the corresponding thresholds
  - Clinical metrics (specificity, sensitivity, etc) are generated on the binarized predictions

At this point, we have 8 sets of predictions, 8 pairs of (AUROC, AUPRC) values, 8*3 thresholds, and 8*3 sets of clinical metrics.

3. We repeat steps 1. and 2. 125 times, which gives us 125 * 8 = 1000 sets of predictions, 1000 pairs of (AUROC, AUPRC) values, 1000*3 thresholds, and 1000*3 sets of clinical metrics. We calculate the empirical 95% confidence intervals by taking the 2.5th and the 97.5th percentiles, i.e. the 25th and the 975th values from the sorted 1000 e.g. AUROC values as lower and upper bounds, respectively.

### Large scale validation of machine-read OCT features for diagnosis

As in the prediction task, the logistic regression framework was applied to diagnose the current exudative AMD status of each OCT B-scan volume (14615 OCT volumes, 4182 patients). In this analysis, two feature sets were compared: (1) EHR-derived risk factors (age, Smoking Status, Race, Ethnicity, Sex, and Chronic comorbidities), and (2) EHR-derived risk factors and machine-read structural OCT B-scan AMD risk factors (hcDVh, IHRF, hDC, SDD, and RPD). Model performance was quantified in terms of area under the receiver operating characteristic curve (AUROC) and area under the precision-recall curve (AUPRC).

### Considering AMD status of fellow eye as a feature

In order to investigate the added value of the status of the fellow eye, we ran the following experiment. For each feature group (current AMD status, EHR baseline, biomarkers, combined) the predictive capabilities of the models built on them with and without the fellow eye status were compared.

One exam can be identified uniquely by the PatientID, Laterality, and exam_date. The feature fellow_eye was added to each exam entry by finding the wetAMD value (binary) in the entry with the same PatientID, same exam_date, but opposite Laterality. If no such an entry was found, non-presence of wetAMD was imputed—since in case of even suspected disease progression, an examination would have been performed, which would have been reflected as an entry.

The added utility of the feature was quantified by means of differences in AUROC and AUPRC compared to the corresponding models without fellow eye AMD status.

### Ethics statement

The retrospective imaging data analysis was approved by the UCLA Institutional Review Board (IRB) #15–000083 –Ocular Imaging studies. As the data collection was retrospective, a waiver of informed consent was granted. The research was conducted in accordance with the tenets set forth in the Declaration of Helsinki. All imaging data were transferred to the Doheny Image Reading Research Laboratory (DIRRL) in a de-identified fashion and the imaging analysis was performed.

## Supporting information

S1 Fig Model weights for biomarkers in the fitted *biomarkers* model. Black error bars indicate standard deviation of values obtained by fitting the model to bootstrapped subsets of the cohort.
(TIF)

S2 Fig Heatmap representing correlations between features. (TIF)

S3 Fig Bar plot depicting performances of 2-year predicting models with and without fellow eye status as a predictor. (TIF)

S4 Fig Performance of models built for diagnosis of wet AMD expanded with the AMD status of the fellow eye. (TIF)

S5 Fig Prediction of exudative AMD Conversion. For every week on the x-axis a separate model was trained and evaluated on for the corresponding time frame. *Left*. Area under the ROC curve (AUROC) as a function of prediction time frame. *Right*. Area under the Precision-Recall curve (AUPRC) as a function of prediction time frame. 95% Confidence intervals were computed using bootstrapping.
(TIF)
