# Supplementary figures and images for: Automated large-scale prediction of exudative AMD progression using machine-read OCT biomarkers
Source: PLOS Digit Health. 2023 Feb 15;2(2):e0000106. doi: 10.1371/journal.pdig.0000106 (PMC9931262; doi:10.1371/journal.pdig.0000106)

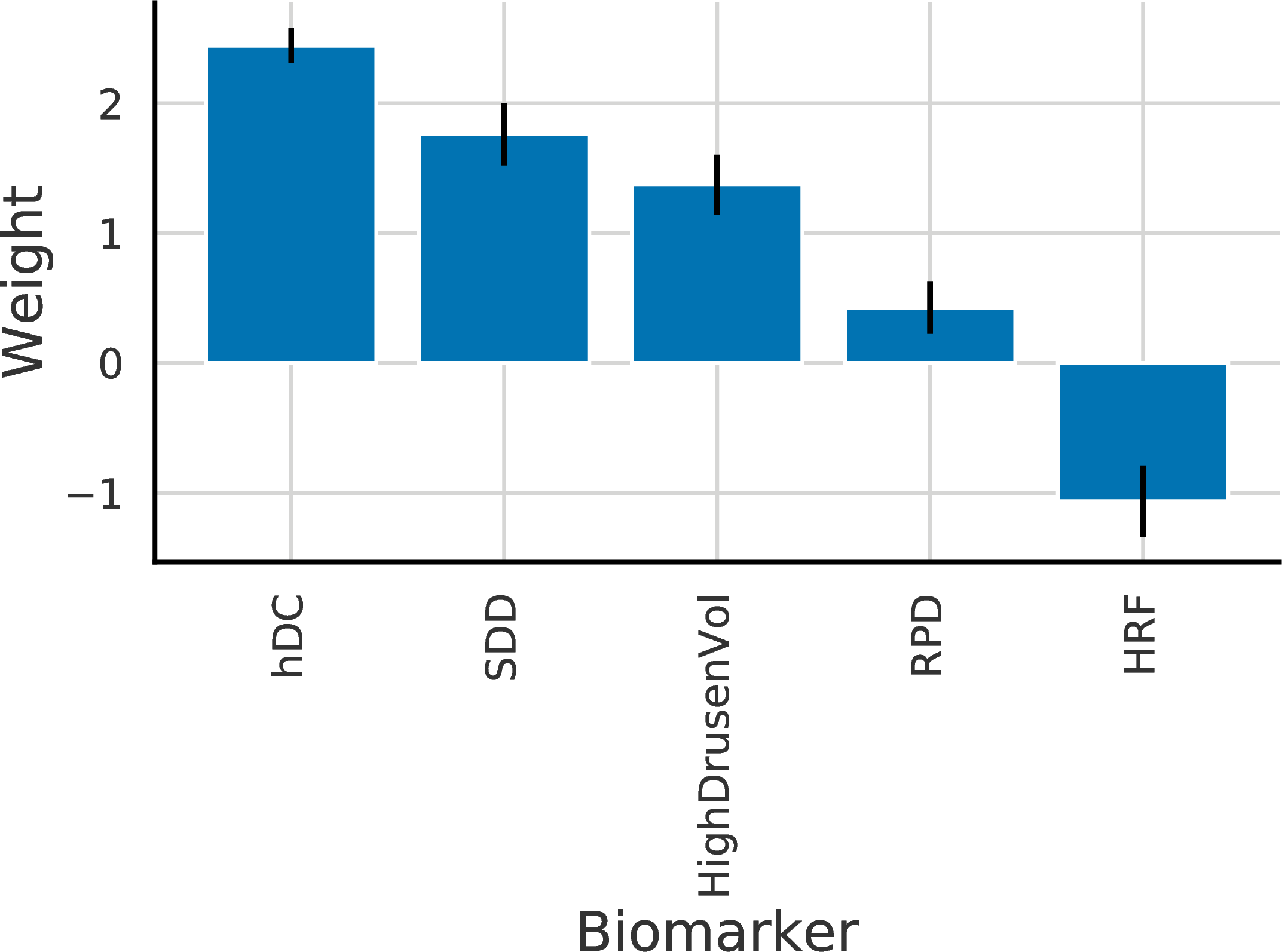

Supplement: S1 Fig — Black error bars indicate standard deviation of values obtained by fitting the model to bootstrapped subsets of the cohort. (TIF) [file pdig.0000106.s001.tif]

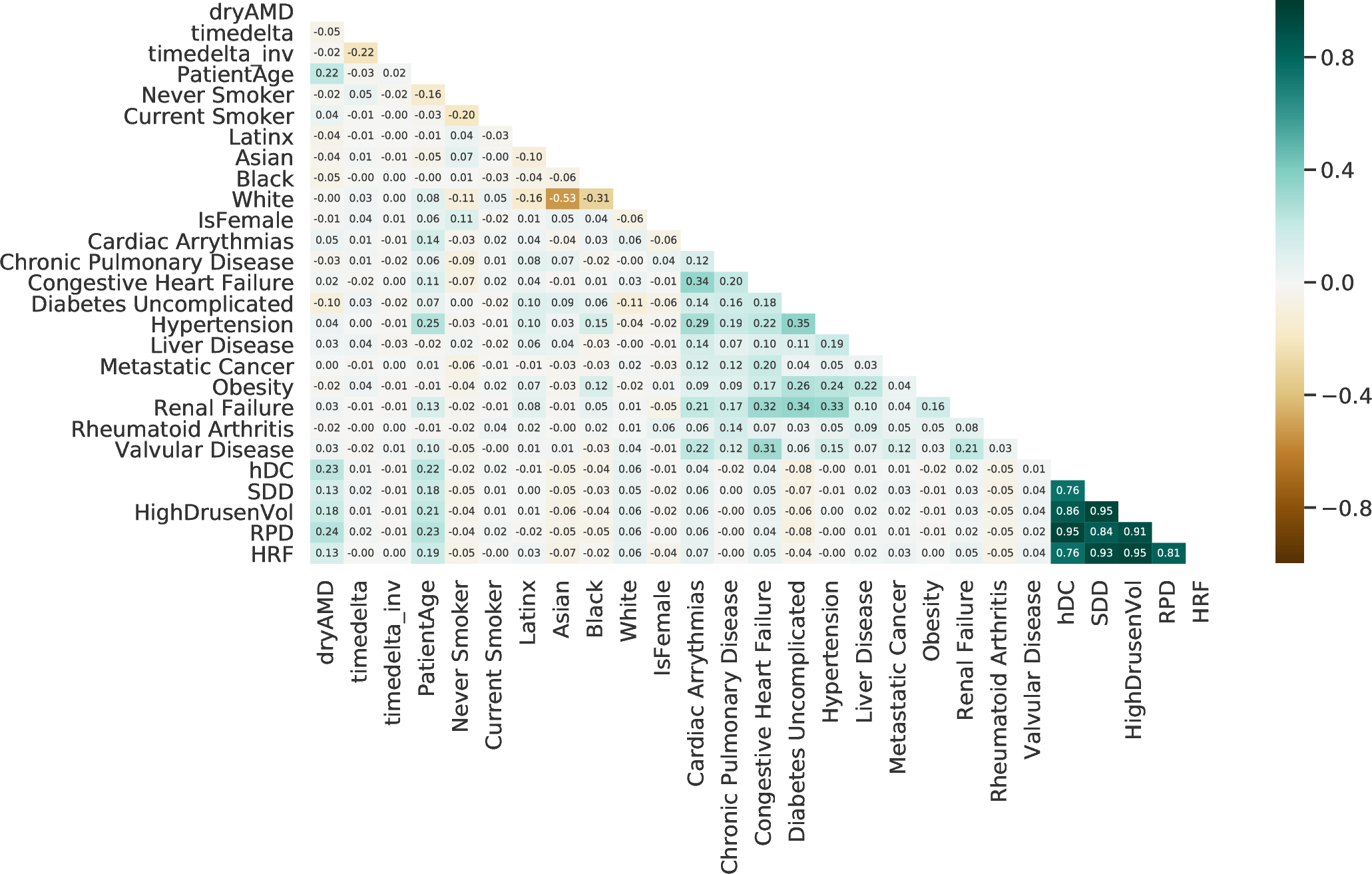

Supplement: S2 Fig — (TIF) [file pdig.0000106.s002.tif]

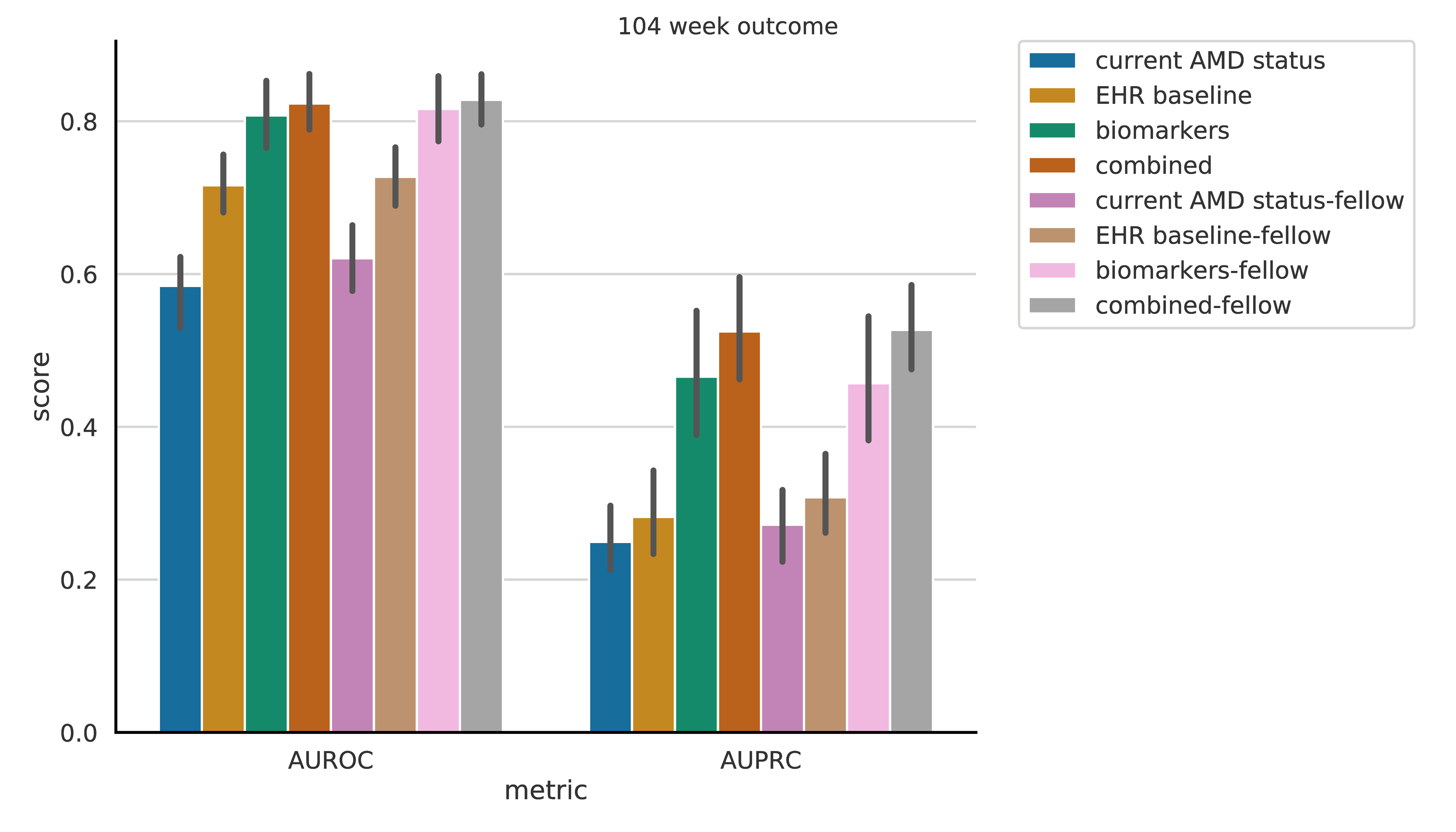

Supplement: S3 Fig — (TIF) [file pdig.0000106.s003.tif]

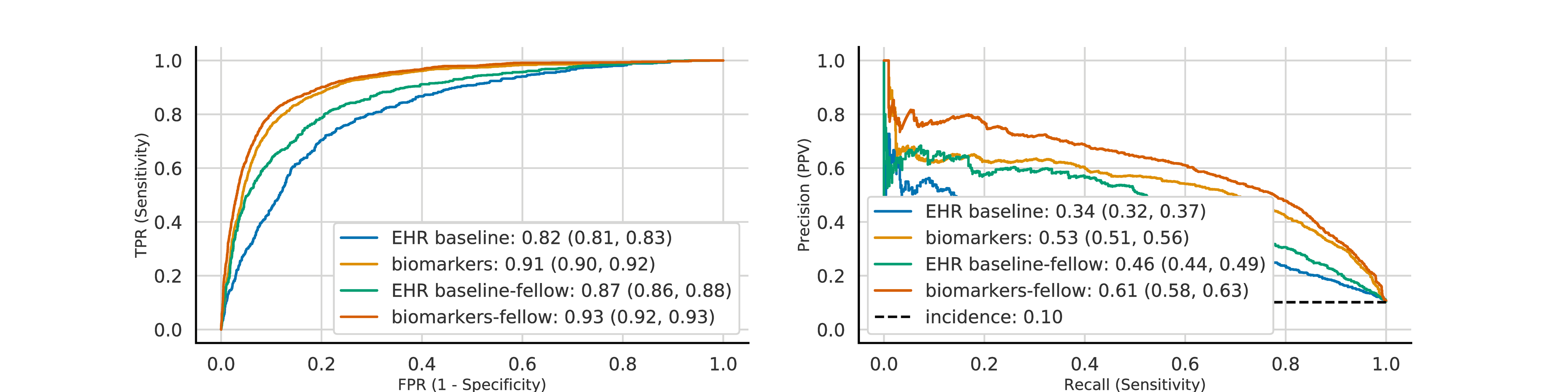

Supplement: S4 Fig — (TIF) [file pdig.0000106.s004.tif]

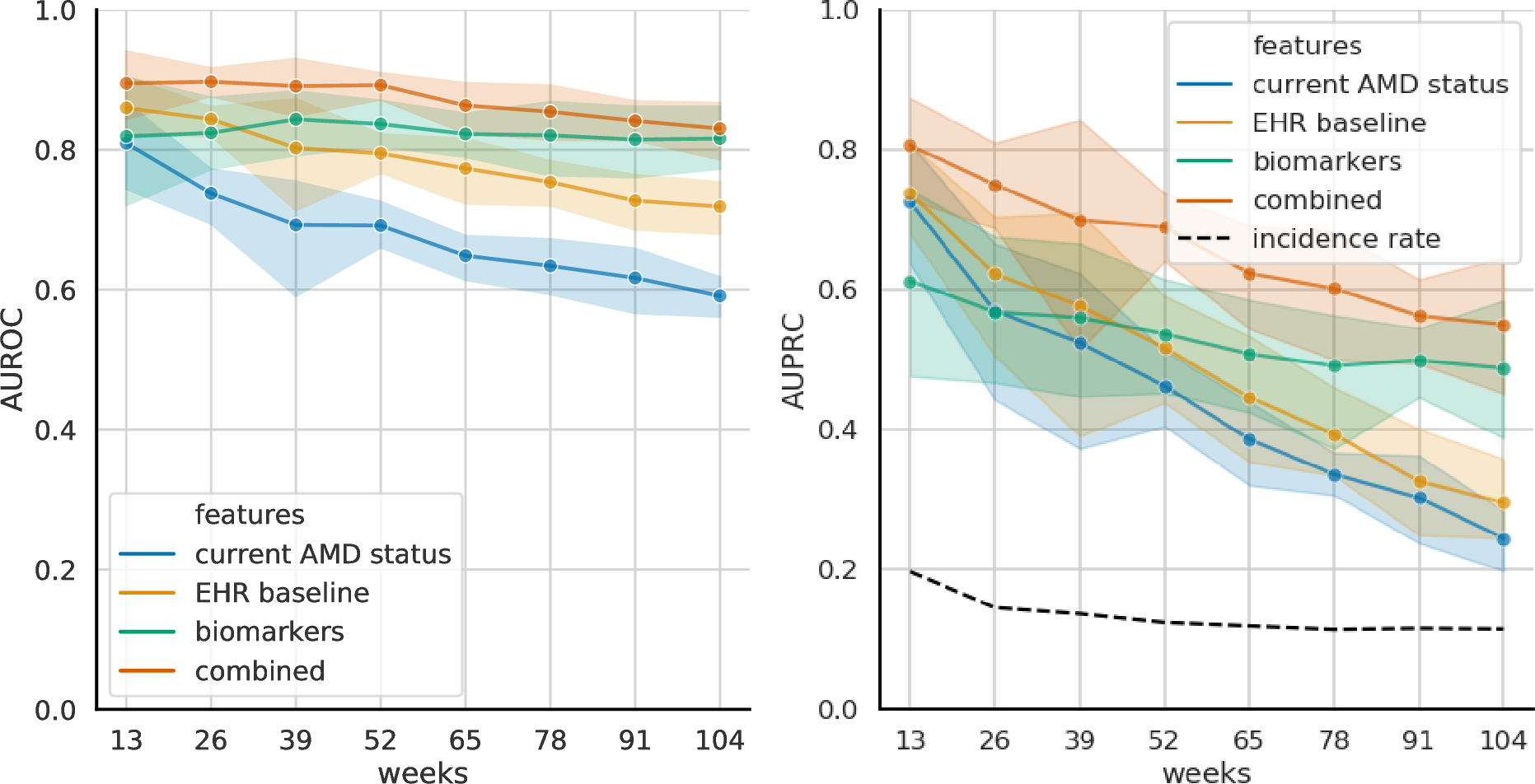

Supplement: S5 Fig — For every week on the x-axis a separate model was trained and evaluated on for the corresponding time frame. Left. Area under the ROC curve (AUROC) as a function of prediction time frame. Right. Area under the Precision-Recall curve (AUPRC) as a function of prediction time frame. 95% Confidence intervals were computed using bootstrapping. (TIF) [file pdig.0000106.s005.tif]
